# Assessing Fluid Resuscitation in Adults with Sepsis Who Are Not Mechanically Ventilated: a Systematic Review of Diagnostic Test Accuracy Studies

**DOI:** 10.1007/s11606-019-05073-9

**Published:** 2019-05-31

**Authors:** Adam Seccombe, Lauren McCluskey, Hannah Moorey, Daniel Lasserson, Elizabeth Sapey

**Affiliations:** 1 Birmingham Acute Care Research Group, University of Birmingham, Birmingham, UK; 2 Worcestershire Acute Hospitals NHS Trust, Redditch, UK

**Keywords:** sepsis, fluid assessment, fluid responsiveness, intravenous fluid, acute medicine

## Abstract

**Background:**

Fluid resuscitation is a widely used intervention that is mandated in the management of sepsis. While its use can be life-saving, its overuse is associated with harm. Despite this, the best means of assessing a need for fluid resuscitation in an acute medical setting is unclear.

**Objective:**

To assess studies of diagnostic tests that identify the need for fluid resuscitation in adults with sepsis, as defined by the presence of fluid responsiveness.

**Design:**

Protocol registration was performed in advance (PROSPERO:CRD42017048651). Research database searches were performed alongside additional searches to identify grey literature. Diagnostic test accuracy studies that assessed any fluid assessment tool were identified independently by two authors, before data extraction and quality assessments were performed.

**Participants:**

Adults with sepsis, without intensive care organ support, who would be appropriate for admission to an acute medical unit.

**Key Results:**

Of the 26,841 articles that were screened, 14 studies were identified for inclusion, involving a combined total of 594 patients. Five categories of index test were identified: inferior vena cava collapsibility index (IVCCI), haemodynamic change with passive leg raise, haemodynamic change with respiration, haemodynamic change with intravenous fluid administration, and static assessment tools. Due to the high level of clinical heterogeneity affecting all aspects of study design, quantitative analysis was not feasible. There was a lack of consensus on reference tests to determine fluid responsiveness.

**Conclusion:**

While fluid resuscitation is considered a key part of the management of sepsis, evidence to support fluid assessment in awake adults is lacking. This review has highlighted a number of research recommendations that should be addressed as a matter of urgency if patient harm is to be avoided.

## INTRODUCTION

Fluid resuscitation is integral to the management of the hypovolaemic, acutely unwell patient,^1^ but assessing whether patients require intravenous fluid is complex as multiple factors can affect fluid balance.^2^ The process currently relies on recognising clinical features of hypovolaemia and hypervolaemia but these features are non-specific.^3^ While competence in fluid assessment is a training requirement for all grades of clinicians,^4^ preventable morbidity due to intravenous fluid mismanagement is commonly identified, with one in five patients suffering complications.^5^ An effective assessment tool must strike a balance that limits such harms without impacting upon or delaying the life-saving benefits of fluid resuscitation.

The National Institute of Health and Care Excellence (NICE) intravenous fluid guidelines^5^ for the UK advocate fluid assessment using a combination of clinical features and bedside tests. Although there is no formal definition of the term “hypovolaemia”, the guidelines list an approach to its identification. However, all but one of the suggested measures are non-specific and two, respiratory rate and the National Early Warning Score,^6^ do not reflect fluid status but simply identify acutely unwell patients. Of note, the NICE guideline development group stated that their recommendations were based on consensus opinion, extrapolated from systematic reviews within older guidelines^7^ that focused on acute illness rather than hypovolaemia. There are no equivalent guidelines from American or European healthcare bodies.

Fluid assessment is particularly challenging in sepsis as haemodynamic compromise is primarily caused by three different factors: vasodilatation, increased vascular permeability, and cardiac dysfunction. Ensuring the dose of IV fluid is optimised is important as excess intravenous fluid has been shown to cause harm in patients with sepsis,^8–10^ with further trials ongoing.^11, 12^ The Surviving Sepsis guidelines advise a dose of fluid over the first 3 h equating to > 2 L in a 70-kg patient.^13^ Following this initial dose, they recommend additional fluid use is guided by frequent reassessment of hemodynamic status. However, they are unable to specify how this should be performed. Furthermore, they acknowledge that these recommendations are supported by a low quality of evidence.^13^

Over the last two decades, studies and systematic reviews^14–17^ have focused on the ability of fluid assessment tools to predict fluid responsiveness: a haemodynamic improvement following fluid bolus. It is a dynamic test, i.e. describes change over two measurements, in contrast to static tests that use a single measurement. The presence of fluid responsiveness can be estimated through indirect means such as performing a passive leg raise^18^ which increases cardiac venous return, or by observing changes in measurements during respiration.^19^

The vast majority of published studies of fluid responsiveness are based in the intensive care unit (ICU), using patients who are sedated and ventilated. Because differences in accuracy between mechanically ventilated and non-mechanically ventilated patients have been observed,^20^ these findings may not translate to acute medical patients.

This systematic review assessed studies of adults with sepsis who would be appropriate for admission to the acute medical unit (AMU) and compared index tests designed to determine the need for fluid resuscitation with reference standards that identify the presence of fluid responsiveness.

## METHODOLOGY

This systematic review was reported using PRISMA (Preferred Reporting Items for Systematic Reviews and Meta-analyses) guidelines.^21^ Prior to commencing the review, the protocol was published (PROSPERO:CRD42017048651).^22^

### Search Strategy

An electronic database search was undertaken up to 14 June 2018, following consultation with an information specialist, using keywords and subject headings that encompassed three domains: sepsis, intravenous fluid, and patient location. Search strategies were piloted prior to use. Appendix 1 (online) contains a sample search strategy for MEDLINE. The following bibliographic databases were searched: MEDLINE (Ovid) from 1946; Embase (Ovid) from 1947; CINAHL (Ebsco) from 1937; and the Cochrane Library (Wiley) from 1996. No restrictions on publication language or date were applied.

References of included articles and relevant systematic reviews were hand-searched, forward citation searching of included articles was performed via Web of Science, and simplified search strategies were completed in the Zetoc database (The British Library) and the Conference Proceedings Citation Index (Web of Science) to identify grey literature. A search of research registers (ClinicalTrials.gov, UK Clinical Research Network Study Portfolio Database, and World Health Organization International Clinical Trials Registry Platform) was undertaken to identify relevant, ongoing studies.

Results from these searches were entered into reference management software (Endnote version 7.3.1 [Clarivate Analytics, Philadelphia, PA, USA]). Duplicate records were removed using an automated algorithm and subsequent manual searching.

### Study Selection

Studies were considered for inclusion based on pre-defined eligibility criteria (Appendix Table 5, online). In brief, these were diagnostic test accuracy studies incorporating an index text used to assess hypovolaemia or the need for fluid resuscitation, including adults with sepsis who were not sedated or anaesthetised. If a study included multiple presenting diagnoses, the authors were contacted to obtain data for patients with sepsis. If > 50% of participants had sepsis or confirmed infection, the study was included regardless. As per the protocol, studies set in ICU were included because of a paucity of evidence. During the search process, it was noted that the majority of studies did not report the proportion of patients who were sedated or anaesthetised. Therefore, the eligibility criteria were changed to exclude patients who were mechanically ventilated.

An initial screening process was completed independently by two reviewers using titles and abstracts. Selected studies were reviewed in full according to the above criteria. Disagreements were resolved through discussion, supported by a third reviewer. Results were recorded using Microsoft Excel 2010 (Microsoft Corporation, Redmond, WA, USA).

### Data Extraction and Evidence Synthesis

Data were extracted using a piloted, standardised form, following translation of non-English language articles if required. The following data were extracted: study characteristics (including setting and sample size); patient characteristics (including age, gender, acuity score, blood pressure, heart rate, preceding intravenous fluid volume, concurrent use of vasopressors/inotropes, admission diagnoses); and details of the index test(s), reference standard, and target condition. Study authors were contacted directly to request missing data. Study quality and risk of bias were assessed using a modified version of QUADAS-2.^23^ The heterogeneity of study design and outcomes precluded a meta-analysis of quantitative results, so a narrative overview of the studies is presented.

## RESULTS

**Figure 1:**
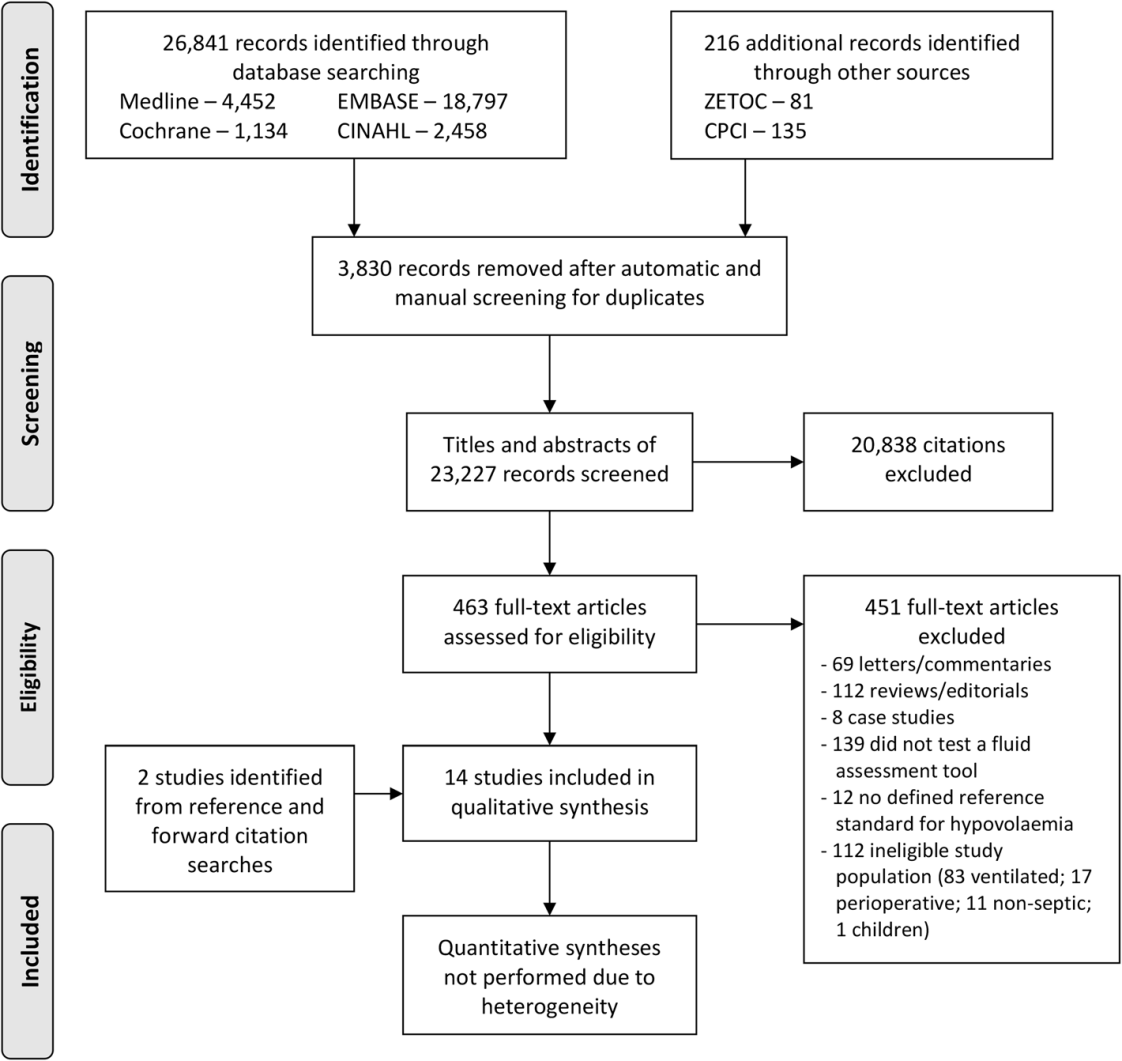
**Flowchart of study selection. Legend: flowchart summarising study selection and inclusion processes in this systematic review, including the reasons for exclusion of all full-text articles that were reviewed.**

Searches returned 26,841 records from the four databases. Two hundred sixteen records were identified from conference proceedings databases and trial registries. Following screening by title and abstract, 463 full-text articles were reviewed. Of the 124 that were diagnostic test accuracy studies, 83 were excluded because the study population was ventilated. Fourteen articles were identified for inclusion.^24–37^ Figure 1 details the selection process and reasons for exclusion.

### Study Characteristics

The 14 included studies had a median sample size of 33 (range 14–116) and were primarily single-centre. Three were published as a conference abstract and two were set outside the ICU, both in emergency departments (ED).

Nine studies based inclusion on the presence of a composite definition of shock, most commonly referred to as acute circulatory failure, defined by the presence of one or more haemodynamic markers, clinical signs, and blood tests. Blood pressure was the key driver for study inclusion. In 13 studies, patients met the inclusion criteria if they were hypotensive alone (Table 1). The most common reason for patient exclusion was an arrhythmia (including irregular heart rhythm), used in seven studies. Another excluded all patients with any form of cardiac disease.

**Table 1:** Main Characteristics of Included Studies

Author	Year	Setting	No. of patients	Patient population	Primary index test(s)	Additional index test(s)	Reference standard	Target condition	Hypovolaemia mentioned?
de Valk^24^	2014	ED, Netherlands	23*	Shock (SBP < 90, SBP > 40 less than normal, HR > 100, CRT > 2 s, or lactate > 2)	IVCCI	None	Rise in SBP after fluid bolus	Fluid responsiveness	Yes
Corl^25^	2017	ED/ICU, USA	55*	ACF (SBP < 90/MAP < 65 for > 30 min, UO < 0.5, HR > 120 for > 30 min, pH < 7.3, or lactate > 2)	IVCCI	Change in IVCCI after fluid and PLR, IVCDi/e	Rise in CI after fluid bolus	Fluid responsiveness	No
Muller^26^	2012	ICU, France	40	ACF (MAP < 65, UO < 0.5, tachycardia, mottled skin, or lactate > 2)	IVCCI	E wave velocity, LVOT VTI, E/A ratio, E/Ea ratio	Rise in LVOT VTI after	Fluid responsiveness	No
Preau^27^	2017	ICU, France	90	Sepsis and ACF (SBP < 90, SBP > 40 less than normal, UO < 0.5, HR > 100, or mottled skin)	IVCCI ± standardised respiration	IVCD, SVI	Rise in SVI after fluid bolus	Fluid responsiveness	Yes
Lanspa^28^	2013	ED/ICU, USA	14	Sepsis and refractory hypotension (SBP < 90 after > 20 mL/kg of IV fluid)	IVVCI, AoVV, and SVV	None	Rise in CI after fluid bolus	Fluid responsiveness	No
Abodorra^29^ (Poster)	2014	ICU, Egypt	40	Sepsis and ACF (undefined)	IVCCI (after fluid bolus)	Change in IVCCI after fluid	Rise in LVOT VTI after fluid bolus	Fluid responsiveness	No
Dutta^30^ (poster)	2014	ED/ICU, India	116	Sepsis and hypotension (undefined)	Change in SV after PLR	None	Rise in SV after fluid bolus	Fluid responsiveness	Yes
Klarer^31^ (poster)	2010	ICU, Switzerland	27	Hypotension (MAP < 60 mmHg) and/or reduced CI (CI < 2.7 L/min/m^2^)	Change in CI, SVI, and MAP after PLR	None	Rise in CI after fluid bolus	Fluid responsiveness	No
Preau^32^	2010	ICU, France	34	Sepsis or acute pancreatitis and ACF (SBP < 90, SBP > 40 less than normal, UO < 0.5, HR > 100, or mottled skin)	Change in SV, PP, and VF after PLR	None	Rise in SV after fluid bolus	Fluid responsiveness	Yes
Soubrier^33^	2007	ICU, France	32	Haemodynamic instability (SBP < 90, MAP < 75, SBP > 40 less than normal, UO < 0.5 over 3 h, HR > 100, or mottled skin)	PPV and SBPV ± standardised respiration	None	Rise in CI after fluid bolus	Fluid responsiveness	Yes
Preau^34^	2012	ICU, France	23	ACF (SBP < 90, SBP > 40 less than normal, UO < 0.5 for > 1 h, HR > 100, or mottled skin)	PPV and VFV ± standardised respiration	None	Rise in SV after fluid bolus	Fluid responsiveness	No
Jung^35^	2012	ED, South Korea	26	Sepsis and hypotension (SBP < 90, MAP < 70, SBP > 40 less than normal in the absence of another cause)	FTc	CVP, IVCD	Rise in SV after fluid bolus	Fluid responsiveness	No
Keller^36^	2009	ICU, USA	44	Any admission to ICU with a plan to insert a CVC	IJV aspect ratio	None	CVP < 8 mmHg	Fluid responsiveness	No
Soliman^37^	2017	ICU, Egypt	30	Sepsis and hypotension (MAP < 65) or impaired tissue perfusion (lactate > 4)	Change in CO after fluid	None	MAP > 65 and lactate < 4	Fluid responsiveness	No

Summary of the 14 included studies and illustrating the wide heterogeneity. Primary index tests are those mentioned in the study’s aim.

*ACF* acute circulatory failure, *AoVV* aortic velocity variation, *CI* cardiac index, *CO* cardiac output, *CRT* capillary refill time, *CVP* central venous pressure, *ED* emergency department, *FTc* corrected flow time, *HR* heart rate, *ICU* intensive care unit, *IJV* internal jugular vein, *IVCCI* inferior vena cava collapsibility index, *IVCDi/e* end-inspiratory/expiratory inferior vena cava diameter, *LVOT VTI* left ventricular outflow tract velocity time integral, *MAP* mean arterial pressure, *PLR* passive leg raise, *PP* pulse pressure, *PPV* pulse pressure variation, *SBP* systolic blood pressure, *SBPV* systolic blood pressure variation, *SV* stroke volume, *SVI* stroke volume index, *SVV* stroke volume variation, *UO* urine output, *VF* femoral artery velocity, *VFV* femoral artery velocity variation

*Individual patient data

### Patient Population

Eight studies exclusively included patients with sepsis. Two authors from the remaining six studies provided individual patient data for septic patients. Of note, patients with an admission diagnosis of heart failure were present in two studies. The recording of preceding intravenous fluid use was limited, mentioned in five studies. In two of these, patients received a median of 4 L prior to participation, double the minimum initial fluid dose for sepsis according to the Surviving Sepsis guidelines^13^ (Table 2).

**Table 2:** Main Patient Characteristics

Author	Year	Male (%)	Age (years)	MAP (mmHg)	HR (bpm)	Diagnoses	Additional treatment (e.g. inotropes)	Preceding IV fluid (L)
de Valk^24^*	2014	23 (48)	55 ± 18	75 ± 15	117 ± 8	Sepsis (100)	–	M 100 (Q 0–325)
Corl^25^*	2017	23 (42)	68 ± 19	99 ± 19	115 ± 30	Sepsis (100)	Vasopressors (58)	M 4000 (Q 3350–6000)
Muller^26^	2012	–	M 63 (5P 56, 95P 70)	M 71 (5P 66, 95P 77)	M 101 (5P 91, 95P 116)	Sepsis (60), bleeding (28), dehydration (13)	–	–
Preau^27^	2017	58 (64)	55 ± 29	Unknown	102 ± 33	Sepsis (100)	Vasopressors (16)	(within 24 h) M 1000 (0–2500)
Lanspa^28^	2013	5 (36)	M 62 (Q 46–81)	M 65 (Q 61–70)	M 102 (Q 80–112)	Sepsis (100)	Vasopressors (57)	M 4600 (Q 3000–5900)
Abodorra^29^	2014	–	54 ± 14	58 ± 12	108 ± 12	Sepsis (100)	–	–
Dutta^30^	2014	–	–	Unknown	Unknown	Sepsis (100)	–	–
Klarer^31^	2010	–	M 60 (R 29–82)	M 61 (R 48–104)	M 104 (R 53–145)	Sepsis (52), heart failure (19), respiratory failure (15), other (14)	Vasopressors/inotropes (100)	–
Preau^32^	2010	19 (56)	53 ± 19	77 ± 14	101 ± 22	Sepsis (82), acute pancreatitis (18)	Vasopressors (18)	–
Soubrier^33^	2007	9 (28)	61 ± 13	89 ± 14	103 ± 16	Sepsis (13), pneumonia (75), haematological disease (3), trauma (6), abdominal surgery (3)	Vasopressors (9)	25% received IV fluid in preceding 24 h
Preau^34^	2012	16 (70)	50 ± 5	79 ± 11	104 ± 19	Sepsis (87), acute pancreatitis (13)	–	–
Jung^35^	2012	17 (65)	M 74 (Q 58–83)	M 57 (Q 50–66)	94 (83–114)	Sepsis (100)	No	–
Keller^36^	2009	22 (50)	66 ± 14	67 ± 12	92 ± 22	Sepsis (46), GI bleed (14), heart failure (9), not recorded (32)	–	–
Soliman^37^	2017	43.3	48 ± 20	53 ± 8	–	Sepsis (100)	Vasopressors (not recorded)	–

Summary of the patient characteristics for the included studies. “–” means data not available. Data are presented as means ± SD or as medians (indicated by “M”) with a measure of spread in brackets (preceded by “Q” if quartiles, “R” if range, and “5P” or “95P” if 5th and 95th percentiles respectively). Number of patients, names of diagnoses, and use of vasopressors are presented with percentages in parenthesis.

*Individual patient data

### Target Condition

The target condition for all studies was fluid responsiveness. However, its relationship to hypovolaemia was poorly articulated. Nine studies failed to use the term “hypovolaemia” at any point in the rationale, methods, or interpretation of findings (Table 1).

### Reference Standard

Table 3 shows the significant heterogeneity in the reference standard, the test used to determine the presence or absence of fluid responsiveness. Only two studies used the same parameter.^32, 34^ There was marked variation in the proportion of patients meeting the reference standard: median 50% (range 17.4–65.4%).

**Table 3:** Summary of Reference Standards

Author	Year	Met reference standard (%)	Measurement			Fluid bolus		
			Parameter	Threshold rise (%)	Measurement tool	Volume	Fluid type	Rate (min)
de Valk^24^	2014	17.4	Systolic blood pressure	> 10 mmHg	NIBP	500 mL	0.9% saline	15
Corl^25^	2017	56.4	Cardiac index	> 10	Bioreactance	500 mL	0.9% saline	Pressure bag
Muller^26^	2012	50	LVOT VTI	> 15	Echocardiography	500 mL	6% starch	15
Preau^27^	2017	55.6	Stroke volume index	> 10	Echocardiography	500 mL	4% gelatine	30
Lanspa^28^	2013	35.7	Cardiac index	> 15	Echocardiography	10 mL/kg	Crystalloid	< 20
Abodorra^29^	2014	50	LVOT VTI	> 15	Echocardiography	500 mL	Not recorded	15
Dutta^30^	2014	62.9	Stroke volume	> 10	Echocardiography	30 mL/kg	Crystalloid	Not recorded
Klarer^31^	2010	Not recorded	Cardiac index	> 15	Pulse contour analysis	500 mL	0.9% saline	15
Preau^32^	2010	41.1	Stroke volume	> 15	Echocardiography	500 mL	6% starch	30
Soubrier^33^	2007	59.4	Cardiac index	> 15	Echocardiography	500 mL	6% starch	20
Preau^34^	2012	43.5	Stroke volume	> 15	Echocardiography	500 mL	6% starch	30
Jung^35^	2012	65.4	Stroke volume	> 10	Oesophageal Doppler	7 mL/kg	6% starch	30
Keller^36^	2009	59.1	Central venous pressure < 8 mmHg via central venous catheter			N/A—static test		
Soliman^37^	2017	33.3	MAP < 65 mmHg or lactate < 4 mmol/L (measurement tool unclear)			N/A—static test		

Summary of the reference standards used by each study described according to the method of measuring the physiological parameter and, if a dynamic assessment tool was used, the means by which the fluid bolus was given.

*LVOT VTI* left ventricular outflow tract velocity time integral, *NIBP* non-invasive blood pressure

Two studies used static tests to identify fluid responsiveness, both from the Surviving Sepsis guidelines.^13^ The remaining 12 used a dynamic test. One study used absolute (> 10 mmHg) rise in non-invasive systolic blood pressure. Eleven studies used a relative rise in cardiac function; the most common measurements were cardiac index and stroke volume (SV), used by four studies each. The most common measurement tool was echocardiography, used in eight studies.

Of the 12 studies that used a dynamic test, all used a fluid bolus. Nine gave a fixed 500 mL bolus. The other three gave a weight-based bolus that ranged between 490 and 2100 mL for a 70-kg patient. The rate of the bolus ranged between 15 and 30 min, although one study gave 500 mL using a pressure bag and noted a variable infusion rate. Five studies used crystalloid, six used colloid, and one did not specify the type of fluid.

### Index Tests

Five categories of index test (i.e. the diagnostic test under evaluation) were identified in the studies and are summarised in Table 4. They included inferior vena cava (IVC) measurements, change following passive leg raise (PLR), change with respiration, change following intravenous fluid, and static measurements.

**Table 4:** Summary of Studied Index Tests

Category of index test	Author	Year	Primary index tests (measurement tool)	Threshold	AUROC	Sn	Sp	PPV	NPV
Inferior vena cava	de Valk^24^*	2014	IVCCI (US)	≥ 36.5%	0.68 (0.37–0.98)	75	57.9	27.3	97.7
	Corl^25^*	2017	IVCCI (US)	≥ 25%	0.82 (0.68–0.95)	83.9	79.2	83.9	79.2
	Muller^26^	2012	IVCCI (US)	≥ 40%	0.77 (0.60–0.88)	70	80	77.8	72.7
	Preau^27^	2017	IVCCI (US)	≥ 48%	0.82 (0.73–0.91)	76	88	88	75
			IVCCI with standardised respiration (US)	≥ 31%	0.89 (0.82–0.97)	84	90	91	82
	Lanspa^28^	2013	IVCCI (US)	≥ 15%	0.83 (0.58–1.00)	100	67	62	100
	Abodorra^29^	2014	IVCCI after 100 mL (US)	> 45%	0.91	90	65	72	88.7
PLR	Dutta^30^	2014	Change in SV (Echo)	≥ 15%	–	87.7	100	100	82.7
	Klarer^31^	2010	Change in CI (PC)	> 15%	–	–	–	50	86
			Change in SVI (PC)	> 15%	–	–	–	20	77
			Change in MAP (PC)	> 10%	–	–	–	12	80
	Preau^32^	2010	Change in SV (Echo)	≥ 10%	0.94 (0.90–0.98)	86	90	86	90
			Change in PP (PC)	≥ 9%	0.86 (0.78–0.94)	79	85	79	85
			Change in VF (Echo)	≥ 8%	0.93 (0.89–0.97)	86	80	75	89
Respiration	Lanspa^28^	2013	AoVV (Echo)	≥ 25%	0.67 (0.32–1.00)	75	66.7	50	85.7
			SVV (PC)	≥ 17%	0.92 (0.73–1.00)	60	100	100	81.8
	Soubrier^33^	2007	PPVa (PC)	≥ 12%	0.81 (0.73–0.89)	63	92	92	63
			SBPV (PC)	≥ 9%	0.82 (0.74–0.90)	47	92	90	54
			PPVa with standardised respiration (PC)	≥ 33%	0.72 (0.63–0.81)	21	92	80	44
			SBPV with standardised respiration (PC)	≥ 30%	0.69 (0.59–0.79)	26	92	80	83
	Preau^34^	2012	PPVa (PC)	≥ 10%	0.71 (0.59–0.83)	60	100	100	76
			VFV (US)	≥ 10%	0.74 (0.63–0.85)	60	100	100	76
			PPVa with standardised respiration (PC)	≥ 12%	0.95 (0.90–1.00)	90	100	100	93
			VFV with standardised respiration (US)	≥ 12%	0.95 (0.90–1.00)	90	100	100	93
Static	Jung^35^	2012	FTc (oesophageal Doppler)	< 301 ms	0.87 (0.71–0.98)	88.2	88.8	93.7	79.9
	Keller^36^	2009	IJV aspect ratio (US)	< 0.83	0.84 (0.72–0.96)	78	77	83	71
Fluid	Soliman^37^	2017	Change in CO (bioimpedance) after 30 mL/kg 0.9% saline over 2 h	> 12.5%	0.9	90	70	80	90

Summary of the primary index tests for included studies. “–” means data not available.

*AoVV* aortic velocity variation, *AUROC* area under the curve of the receiver operating characteristic, *CI* cardiac index, *CO* cardiac output, *FTc* corrected flow time, *IJV* internal jugular vein, *IVCCI* inferior vena cava collapsibility index, *MAP* mean arterial pressure, *NPV* negative predictive value, *PC* pulse contour analysis, *PLR* passive leg raise, *PP* pulse pressure, *PPV* positive predictive value, *PPVa* pulse pressure variation, *SBP* systolic blood pressure, *SBPV* systolic blood pressure variation, *Sn* sensitivity, *Sp* specificity, *SV* stroke volume, *SVI* stroke volume index, *SVV* stroke volume variation, *US* ultrasound, *VF* femoral artery velocity, *VFV* femoral artery velocity variation

*Individual patient data

Six studies assessed inferior vena cava collapsibility index (IVCCI), calculated using the difference in IVC diameter during respiration divided by end-expiratory IVC diameter. Four studies measured IVCCI before a fluid bolus was given and used a measure of left ventricular function to identify fluid responsiveness. The area under the curve of the receiver operating characteristic (AUROC) for IVCCI was similar for each of these studies (0.77, 0.82, 0.82, 0.83). A fifth used systolic blood pressure to identify fluid responsiveness, giving an AUROC of 0.68. The final study looked at IVCCI after a fluid bolus was given and reported an AUROC of 0.91.

Three studies, two published as conference abstracts only, explored haemodynamic change after a PLR. Three separate studies reported haemodynamic change during respiration. A number of different haemodynamic measurements were explored in both categories with SV and pulse pressure the most common. Two studies explored a static tool as part of their primary aim, although others included results for additional index tests (Table 1). Finally, one study assessed haemodynamic change after a fluid bolus, the reference standard in 12 of the other included studies.

### Risk of Bias

**Figure 2:**
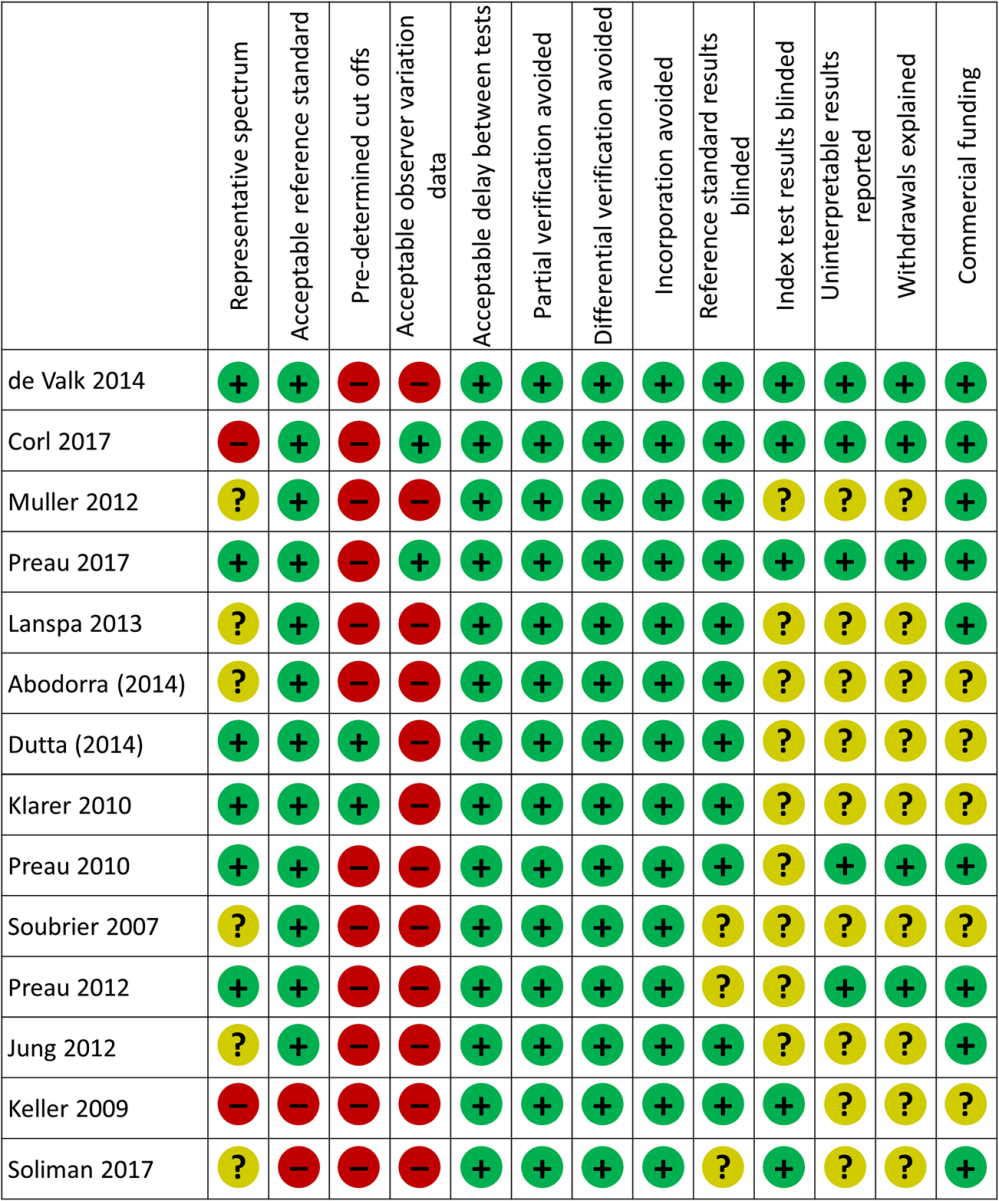
**Risk of bias assessment. Legend: table summarising a risk of bias assessment performed using a modified version of QUADAS-2.** ^23^ **“+”: low risk of bias; “?”: unclear risk of bias; “-”: high risk of bias.**

Several factors contributed to a high overall risk of bias (see Fig. 2). Two studies used static measurements as reference standards (central venous pressure^35^ and blood pressure/lactate levels^36^) whilst acknowledging they would not effectively identify fluid responsiveness. This would bias estimates of sensitivity and specificity. The remaining 12 used fluid responsiveness; however, none provided satisfactory evidence to support their choice of reference standard.

Twelve studies failed to report acceptable observer variation data: nine provided no data for the index test, one reported intra-observer variation only, one reported observer variation in a healthy cohort, and one reported inter-observer variation greater than the index test’s threshold values. Twelve studies calculated the index test’s optimal threshold using post hoc analysis with no a priori definition of a diagnostic cut-off. As a result, in the six studies assessing IVCCI, thresholds ranged from > 15 to >50% (median > 37%). Finally, only five studies reported the proportion of patients with uninterpretable results: three for echocardiography (11.5%, 12.8%, and 16.1%) and two for IVCCI (12.5%, 13.5%).

## DISCUSSION

This is the first systematic review which explores diagnostic tests that determine the need for fluid resuscitation in adults with sepsis who are *not* mechanically ventilated. Despite this, all but two studies were based solely in ICU as opposed to a ward or AMU, where the majority of medical patients are managed.

The characteristics of included patients varied between studies and were often poorly described. Six studies combined septic shock with shock arising from very different pathophysiologies, and seven excluded patients with arrhythmia without justification. This exclusion criterion is especially relevant given our ageing population and the increased prevalence of arrhythmia in this cohort (up to 17% of adults > 80 years have atrial fibrillation^38^).

The most common inclusion criterion was hypotension, which was enough to warrant inclusion by itself in 13 studies. All studies identified fluid responsiveness as the target condition, which was universally seen as equivalent to a benefit from fluid resuscitation.

Fluid responsiveness, the target condition in all studies, was dynamically assessed in 12 studies. However, it was defined in 11 different ways, including variations in the delivery of the fluid bolus, the threshold value, and the measurement tool. A direct measure of cardiac function was used in 11 studies and was assessed by echocardiography in eight studies.

Five categories of index test were described. However, the small size, risk of bias, and clinical heterogeneity of included studies prevented meaningful comparisons. Index test thresholds were chosen post hoc in all but two studies; 12 studies failed to report adequate observer variability data; and none of the included studies specified how their index test should be used in practice, i.e. replace, be added to, or triage for the current diagnostic process. Comparisons were further limited by the inadequate reporting of patient characteristics which impact upon the accuracy of the included index tests. Increases in respiratory rate and tidal volume, frequently seen in critically ill patients, have been shown to affect the IVCCI and respiration variation measurements, for example.^39^

In summary, there was a small number of heterogeneous studies available to guide the provision of intravenous fluid in adults with sepsis who are not mechanically ventilated. This heterogeneity, and a lack of consensus regarding associated definitions, is found throughout the literature.

Given that fluid responsiveness is widely assumed to be the most effective way of guiding fluid resuscitation, a single measurement strategy with a pre-defined threshold would be of great benefit to the medical community. Currently, however, the heterogeneity surrounding the definition of fluid responsiveness limits our ability to learn from previous research and plan for future studies. Others have noted such heterogeneity^40^ and have recognised the need for a consensus definition.^41^ Cardiac output is widely regarded as the best means of determining fluid responsiveness^42^ and is supported by Starling’s principles.^43^ However, its use makes the assumption that an increase in cardiac output is always beneficial. In addition, the ability of cardiac output rises to predict benefit from fluid resuscitation has never been tested against more commonly used haemodynamic markers (e.g. blood pressure) and there are concerns about the practicalities of monitoring cardiac output in a medical ward.

Despite excluding mechanically ventilated patients, the eligibility criteria of this systematic review (Appendix Table 5, online) allowed inclusion of studies that are more limited in their generalisability to an acute medical population. Most studies were set in ICU and many participants had been treated for days before inclusion. The absence of patients who are not critically unwell would have artificially increased the sensitivity of all index tests.^44^ This risk was acknowledged in the protocol^22^ after scoping identified a lack of evidence outside of ICU.

The wide clinical heterogeneity meant statistical tests to exclude publication bias were not feasible. However, the identification of three unpublished conference proceedings reflects a robust search strategy and suggests that the low number of included studies is due to limited evidence rather than methodological shortcomings.

It should also be noted that the diagnostic test accuracy methodology itself limits the analysis of the index tests. This methodology assumes a dichotomous status for an individual, e.g. hypovolaemic or not hypovolaemic. In reality, a spectrum exists between euvolaemia and hypovolaemia. All described index tests provide the clinician with continuous data and therefore would support a nuanced analysis. Simplifying measurement tools to provide a binary answer limits a clinician’s ability to optimise fluid provision.

This systematic review has highlighted multiple research opportunities that should be pursued. Given the variety of assumptions surrounding fluid responsiveness and its frequency in the literature, it should be explored as a priority. After observer variability data has been gathered, the proportion of well and acutely unwell adults who are fluid responsive should be assessed using several haemodynamic measurements. Consideration should be given to the impact of factors such as age, disease severity, and comorbidities on these proportions. To ensure feasibility outside of ICU, non-invasive measurement tools should be used.

Appropriately powered observational studies should then examine potential associations between fluid responsiveness and commonly used outcome measures. Fluid responsiveness has been noted in health and may simply be a marker of cardiac function, and so should also be explored as a prognostic indicator. Each approach should be tested in different aetiologies of shock.

To support the integration of these diagnostic tests into clinical practice, an understanding of the current decision-making process is needed, which will clarify the exact purpose of the chosen diagnostic test. Beyond this point, a randomised controlled trial will be able to measure its impact in a clinical setting.

## CONCLUSION

Intravenous fluid remains a central recommendation in guidelines for the management of sepsis (including the Surviving Sepsis guidelines^13^ and NICE CG174^5^) but evidence to support these recommendations is lacking. There is no consensus definition of hypovolaemia. While recent studies have used fluid responsiveness to identify when fluid resuscitation is required, there is no agreement on which reference standard test should define fluid responsiveness. Finally, if fluid responsiveness is identified, there is no evidence to clarify how it should be treated (i.e. which fluid, volume, and rate), or whether treatment is indicated at all.

While this systematic review highlights a lack of evidence, it identifies research recommendations that, if met, would build an evidence base for the provision of fluids in acutely unwell adults outside of ICU. Intravenous fluids are a common treatment across all medical specialities. Prompt administration can be life-saving, but excessive use is associated with patient harm. Research in this field should, therefore, be a priority for the research community.

## Appendix 1. Search strategy for MEDLINE

1. sepsis.ti,ab
2. septic.ti,ab
3. septicaemia.ti,ab
4. septicemia.ti,ab
5. mods.ti,ab
6. multiple organ dysfunction syndrome.ti,ab
7. mof.ti,ab
8. multiple organ failure.ti,ab
9. sirs.ti,ab
10. systemic inflammatory response syndrome.ti,ab
11. exp Systemic Inflammatory Response Syndrome/
12. exp Multiple Organ Failure/
13. **OR/1-12 (196,454 on 7/2/17; Search repeated on 14/6/18)**
14. fluid* ADJ3 replace*.ti,ab
15. fluid* ADJ3 resuscitat*.ti,ab
16. fluid* ADJ3 infus*.ti,ab
17. fluid* ADJ3 administrat*.ti,ab
18. fluid* ADJ3 restor*.ti,ab
19. volume ADJ3 replace*.ti,ab
20. volume ADJ3 resuscitat*.ti,ab
21. volume ADJ3 infus*.ti,ab
22. volume ADJ3 adminstrat*.ti,ab
23. volume ADJ3 restor*.ti,ab
24. intravenous* ADJ3 fluid*.ti,ab
25. IV fluid*.ti,ab
26. colloid*.ti,ab
27. crystalloid*.ti,ab
28. hypertonic solution*.ti,ab
29. hypertonic saline.ti,ab
30. isotonic solution*.ti,ab
31. isotonic saline.ti,ab
32. ringer*.ti,ab
33. hartman*.ti,ab
34. albumin*.ti,ab
35. gelatin*.ti,ab
36. dextran*.ti,ab
37. starch*.ti,ab
38. exp Fluid Therapy/
39. exp Plasma Substitutes/
40. exp Infusions, Intravenous/
41. exp Colloids/
42. exp Hypertonic solutions/
43. **OR/14-42 (489,435 on 7/2/17; Search repeated on 14/6/18)**
44. inpatient*.ti,ab
45. in-patient*.ti,ab
46. patient ADJ3 admiss*.ti,ab
47. patient ADJ3 admit*.ti,ab
48. hospital*.ti,ab
49. intensive treatment unit*.ti,ab
50. ITU.ti,ab
51. intensive care.ti,ab
52. ICU.ti,ab
53. critical care.ti,ab
54. “accident and emergency”.ti,ab
55. emergency department*.ti,ab
56. emergency room*.ti,ab
57. exp Inpatients/
58. exp Hospitalization/
59. exp Intensive Care Units/
60. exp Critical Care/
61. exp Emergency Service, Hospital/
62. exp Hospital Departments/
63. exp Internal Medicine/
64. **OR/44-63 (2,591,320 on 7/2/17; Search repeated on 14/6/18)**
65. **AND/13,43,64 (4231 on 7/2/17; Search repeated on 14/6/18, 221 records between 2017 to Current)**

## Appendix 2

**Table 5:** Study Selection Criteria

Study design	Diagnostic test accuracy studies
Participants	Adults (aged ≥ 18 years) with sepsis of any severity or confirmed infection. Studies were excluded if they involved children, pregnant women, burns patients, trauma patients, perioperative patients, or patients who were mechanically ventilated.
Index test	Any history question, examination technique, or diagnostic test
Reference standard	Any
Target condition	Hypovolaemia or a need for fluid resuscitation
